# Distribution of Gifsy-3 and of Variants of ST64B and Gifsy-1 Prophages amongst *Salmonella enterica* Serovar Typhimurium Isolates: Evidence that Combinations of Prophages Promote Clonality

**DOI:** 10.1371/journal.pone.0086203

**Published:** 2014-01-24

**Authors:** Lester Hiley, Ning-Xia Fang, Gino R. Micalizzi, John Bates

**Affiliations:** Public Health Microbiology Laboratory, Forensic and Scientific Services, Queensland Department of Health, Brisbane, Queensland, Australia; University of Osnabrueck, Germany

## Abstract

*Salmonella* isolates harbour a range of resident prophages which can influence their virulence and ability to compete and survive in their environment. Phage gene profiling of a range of phage types of *Salmonella enterica* subsp. *enterica* serovar Typhimurium (*S.* Typhimurium) indicates a significant level of correlation of phage gene profile with phage type as well as correlation with genotypes determined by a combination of multi-locus variable-number tandem repeat (VNTR) typing and clustered regularly interspaced short palindromic repeats (CRISPR) typing. Variation in phage gene profiles appears to be partly linked to differences in composition of variants of known prophages. We therefore conducted a study of the distribution of variants of ST64B and Gifsy-1 prophages and coincidently the presence of Gifsy-3 prophage in a range of *S.* Typhimurium phage types and genotypes. We have discovered two variants of the DT104 variant of ST64B and at least two new variants of Gifsy-1 as well as variants of related phage genes. While there is definite correlation between phage type and the prophage profile based on ST64B and Gifsy-1 variants we find stronger correlation between the VNTR/CRISPR genotype and prophage profile. Further differentiation of some genotypes is obtained by addition of the distribution of Gifsy-3 and a sequence variant of the substituted SB26 gene from the DT104 variant of ST64B. To explain the correlation between genotype and prophage profile we propose that suites of resident prophages promote clonality possibly through superinfection exclusion systems.

## Introduction

Strains of *Salmonella enterica* serovar Typhimurium may contain a range of temperate phages residing as prophages in their chromosomes. The prophage load of the host bacterium can be very important to its ability to compete and survive in its environment because prophage genomes may contain virulence and fitness factors which become a driving force in the pathogen-host interaction and can lead to emergence of new epidemic clones [1]. Furthermore, low level release of bacteriophage from lysogenised strains may give them a competitive advantage over phage-sensitive strains [2]. The phage type of a strain is likely to reflect the prophage composition of the isolate because the lysis patterns of the phages in the typing panel may be altered by new phage acquisitions or deletions [3]. Knowledge of the prophage composition as well as the underlying genotype of the bacterial host can give clues to the possible phylogenetic origins of newly emerged epidemic strains [2].

According to current theory, bacteriophage genomes are a mosaic consisting of combinations of modules which are exchangeable among members of the bacteriophage population by either homologous or illegitimate recombination events [4]. A comparison of related phage genomes will typically show regions of close sequence identity interrupted by regions of non-identity. These gaps are mainly DNA replacements where the DNA segment in one phage is replaced in another phage by a segment of unrelated DNA that frequently fulfils the same or related function [1].

The ST64B bacteriophage (henceforth termed ST64B_DT64_) induced from *S.* Typhimurium DT64 [3] is described as a genetic mosaic which has acquired significant portions of its genome from sources outside the genus *Salmonella* [5]. ST64B_DT64_ is grouped with P27-like phages [6] but has similarities to lamboid phages. Several studies have made limited comparisons of the ST64B prophages in different *S.* Typhimurium isolates. Figueroa-Bossi and Bossi [7] reported that the ST64B prophage in DT104 showed some sequence differences in the SB21-22 region compared with ST64B from ATCC 14028s and SL1344. Ross and Heuzenroeder [8] reported that the SB26 gene was the least common of targeted ST64B_DT64_ genes in a panel of isolates which included DTs 126, 108, 170, 12 and 12a but not DT104. Hermans et al [9] using primer sets derived from *S.* Typhimurium DT104 strain 7945 showed that some *S.* Typhimurium strains tested positive for presence of ST64B but lacked the internal sequence from the DT104-derived ST64B. Cooke et al [10] used microarray analysis and PCR targeting prophage sequences to differentiate a panel of 23 *S.* Typhimurium isolates, mainly DT104 and DT170. The primers for ST64B were specific for a sequence in the DT104 form of ST64B (henceforth termed ST64B_DT104_) which could not be amplified in ST64B_DT64_ but this was not recognized at the time.

Figueroa-Bossi et al [11] first described the closely related Gifsy-1 and Gifsy-2 lamboid prophages present in both LT2 and ATCC 14028 strains of *S.* Typhimurium and subsequently reported a third type of related lamboid phage in ATCC 14028 which they called Gifsy-3 [12]. Gifsy-1 and Gifsy-2 occur commonly in serovar Typhimurium but Gifsy-3 is found only rarely [13]. Whilst Gifsy-2 is highly conserved within serovar Typhimurium, Gifsy-1 shows extensive strain-to-strain variability within serovar Typhimurium [13], [14]. DT104 NCTC 13348 carries a Gifsy-1-related prophage, Gifsy-1_DT104_, and DT104 is not immune to infection by Gifsy-1 from ATCC 14028 [2]. Gifsy-1 from LT2 ATCC 43971 (Gifsy-1_LT2_) contains the *GipA* moron [1] but Gifsy-1_DT104_ lacks GipA [13]. Gifsy-1 from LT2, ATCC 14028 and SL1344 all have different immunity modules [15]. Gifsy-1_LT2_ has the same immunity module as Gifsy-2, while Gifsy-1 in SL1344 (Gifsy-1_SL1344_) has the immunity of Gifsy-3 from ATCC 14028 (Gifsy-3_14028_).

Phage typing of *S.* Typhimurium according to the scheme of Anderson [16] has been used for many years to further characterise isolates for epidemiological purposes. The phage sensitivity patterns (or phage type) are determined by the properties of the infecting phages, cell surface factors on the isolate and the susceptibility of the typing phage to immunity-type repressors and to various superinfection exclusion systems controlled by residing prophages. Phage type can be altered by the introduction into the bacterial cell of mobile elements particularly bacteriophages [3] and plasmids [17]. The relationships among different phage types are poorly understood.

MLVA (multilocus variable-number-tandem-repeats analysis) typing is technically less demanding than phage typing and has been found to give generally superior discrimination between isolates [18]. It is more informative than phage typing because it allows isolates to be compared genetically at the chromosomal level and possible phylogenetic relationships to be inferred. The Public Health Microbiology Laboratory at Forensic and Scientific Services has used MLVA for typing of all isolates of *S.* Typhimurium since 2006 and has more recently supplemented this with additional tandem repeats and with clustered regularly interspaced short palindromic repeats (CRISPR) analysis (http://crispr.u-psud.fr/crispr/). The combination of two typing methods has allowed recognition of distinct genotypes termed Repeats Groups (RGs) which correlate strongly with single or numbers of phage types. Trials with phage gene profiling of *S.* Typhimurium isolates have also been conducted [19]. These have included PCR tests for five genes found in the phage ST64B_DT64_, namely SB6, SB26, SB28, SB37 and SB46 (CC-5). We noted that some genotypes including the one which included DT104 NCTC 13348 lacked SB26 but had the other ST64B genes while other genotypes had only the SB46 gene. We hypothesised that the isolates missing only SB26 gene had the ST64B_DT104_ prophage. Investigation into why some genotypes had only SB46 gene led to the discovery of a second set of ST64B genes in Gifsy-1_DT104_ but not in Gifsy-1_LT2_. An oblique reference to this phenomenon was later noted in Figure 3 from Lemire et al [13].

There is a need for a more thorough investigation into the existence of variants of ST64B and Gifsy-1 prophages. We conducted a more comprehensive comparison of available prophage sequences in order to find specific sequences which could identify recognized variants and carried out a survey of well characterised isolates to see how variants are distributed amongst the different genotypes of *S.* Typhimurium.

## Results

### Comparison of ST64B Sequences in DT64, DT2 and DT104

**Figure 1:**
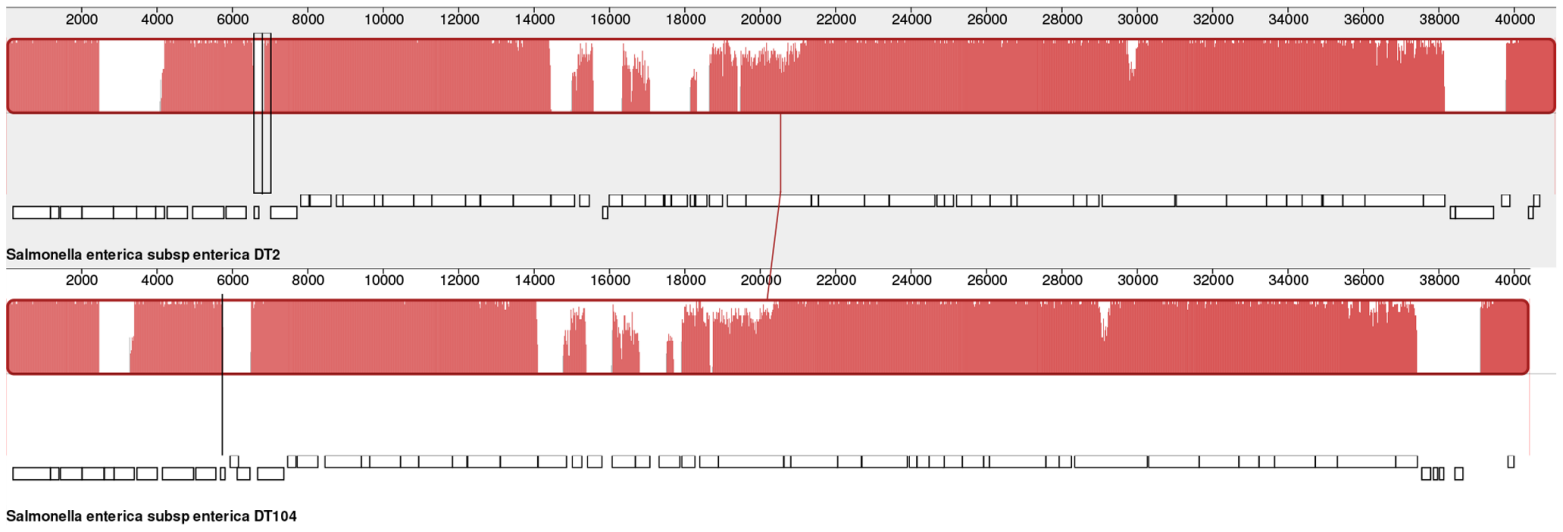
Comparison of ST64B sequences for *S.* Typhimurium strains DT2 (Sanger) and DT104 (NCTC 13348) by MAUVE (See also Figure S3 for gene locations and levels of identity for various regions).

The ST64B sequences from DT64 (ST64B_DT64_), DT2 (ST64B_DT2_) and DT104 (ST64B_DT104_) were 40149, 40147 and 39463 bp respectively in total length. The overall differences between ST64B_DT64_ and ST64B_DT2_ sequences were confined to relatively few single-nucleotide polymorphisms (SNPs) and Indels so that comparison of ST64B_DT104_ with ST64B_DT2_ was essentially the same as with ST64B_DT64_. The comparison of ST64B_DT104_ and ST64B_DT2_ showed that there were six regions of non-identity corresponding to ST64B_DT64_ genes SB32–34, SB37–38, SB49, SB51, SB53–55, SB26+half SB27 and one of only 66% identity (SB52) (Figure S3). A visual comparison of the two ST64B sequences using MAUVE is shown (Figure 1).

### Testing of Isolates for ST64B_DT64_ and ST64B_DT104_ Sequences

#### Distribution of SB26 gene of ST64B_DT64_

We tested for SB26 gene of ST64B_DT64_ in all 214 isolates belonging to the panel of well characterised isolates (Figures S1 and S2). These have been divided into Major Groups 1 and 2 with each Major Group subdivided into numbers of Repeats Groups. We found that SB26 was detected in all but one of the isolates in Major Group 2 but only in two RDNC isolates belonging to Repeats Group 2A (RG2A) in Major Group 1. The DNA sequences of the SB26 allele in these two RDNC strains and for a few controls from isolates in Major Group 2 were the same as for the SB26 allele in ST64B_DT64_. On the basis of the phage gene profiling results [19] and testing for the SB26 gene in the characterised panel of isolates we made the assumption that isolates positive for the SB26 gene probably contain the ST64B_DT64_ prophage.

RG2 (2A+2B) consists of mostly DT4, DT141 and DT141 var 1 and 2 isolates and includes the sequenced strain LT2 ATCC 43971. All members of RG2 have an STTR11 allele of 716 bp rarely seen in isolates of any other Repeats Group. We tested a further 59 isolates which had MLVA types consistent with those of RG2 and the 716 bp STTR11 allele. Eleven isolates tested positive for the SB26 gene and were positive also for SB6, SB28, SB37 and SB46 genes of ST64B_DT64_. The presence of all five genes was taken as presumptive evidence that the whole ST64B_DT64_ prophage was present in these isolates. Further examination showed that all the RG2 isolates with the SB26 gene belonged to RG2A which is distinguished from RG2B on the basis of different CRISPR 2 profiles (Figure S2).

#### Distribution of ST64B_DT104_-specific sequences

We validated the PCR tests for the ST64B_DT104_-specific sequences by testing isolates from RG1 (DT170 and DT12) and RG8 (DT104L and DT12L) which were expected to be positive from phage gene profile results [19] and an isolate from RG13A expected to be negative. RG1 and RG8 isolates were positive for all seven ST64B_DT104_-specific sequences and the RG13A isolate was negative for all seven sequences. All seven PCR products for one isolate from each of RG1 and RG8 were sequenced and found to have 100% identity with the DT104 sequences. This was presumptive evidence that the samples with all seven ST64B_DT104_-specific sequences harboured the whole ST64B_DT104_ prophage.

We subsequently undertook testing of a representative selection of isolates from the various RGs in Major Groups 1 and 2 for all seven ST64B_DT104_ phage sequences. None of the Major Group 2 isolates had any of the ST64B_DT104_ sequences. All of the isolates in RG1 and RG8 were positive for all of the ST64B_DT104_-specific sequences. One of four isolates in RG6A with a 171-x-0-0-462 MLVA profile type was positive for the seven sequences and all four of the isolates in RG6B with a 171-x-x-0-489 MLVA type were positive as well. An isolate typed anomalously as DT126 but with MLVA and CRISPR types consistent with membership of RG2A was positive for all seven ST64B_DT104_ sequences. All isolates in RG9A were positive only for the SB26_DT104_ gene sequence but isolates in RG9B were negative for this sequence. RG9A consists mostly of DT197 isolates with a small number typed as DT6 and DT43, and one, anomalously, as DT104L whereas RG9B consists of DT12a isolates. RG9B is distinguished from RG9A by a significantly different CRISPR 2 profile (Figure S2) although the CRISPR 1 profile is identical to that of RG9A (Figure S1). The SB26_DT104_ gene product from RG9A isolates was found to have a nucleotide sequence which shared only 97% identity with the sequence in ST64B_DT104_. In RG7, two isolates, 05P41568972 typed as DT1 and 09P84391818 as DT41, with the same MLVA and CRISPR profiles, along with ATCC 13311, which had almost the same MLVA profile, were all found to be positive for the ST64B_DT104_ sequences corresponding to SB26 gene, SB33 gene and SB37–38 intergenic sequence and for SB28 and SB37 shared by both types of ST64B prophage but not for the ST64B_DT104_-specific SB49, 50/51 intergenic, SB52 and SB53 sequences and not for SB6 or SB46 common to both ST64B prophages. It appears therefore that these isolates have a variant of ST64B_DT104_ in which the sequence between SB37 to beyond SB6 is altered to some extent.

We wished to clarify the ST64B status of RG6B members with a 171-x-x-0-489 MLVA profile which is shared by members of RG3 (Figures S1 and S2) by testing a larger number of isolates belonging to the two groups. Thirty isolates assigned to RG3 consisting of mostly DT101, DT12a and a few RDNC isolates with an STTR7 allele of 620 bp (except for two with 582 bp allele) and an ST3 allele of 200 bp (except for two with 189 bp allele) were found to be uniformly negative for ST64B_DT104_-specific sequences. Forty isolates assigned to RG6B consisting of DT120 (8), DT193 (12), DT195 (1), U302 (2), RDNC (2) and Untypable (15) isolates, which all had STTR7 alleles of 582 bp and ST3 alleles of 189 bp, were all positive for ST64B_DT104_-specific sequences. It will be shown later in the results section, that the RG3 isolates have a different Gifsy-1 prophage from that in RG6B. It was also noted from the antibiotic sensitivity results that accompany phage typing results that 29 out the 30 isolates in RG3 had a fully sensitive antibiotic profile whereas 38 of the 40 isolates in RG6B had a multi-resistant antibiotic profile most commonly AMP STR TET SUL. Significantly too, clinical notes supplied with the request slips indicated that at least 8 patients with RG6B isolates had a history of travel to SE Asia (7) or Middle East (1) while there was no travel history for any patients with the RG3 isolates. Furthermore, RG6B isolates had not been seen prior to 2006 and the numbers have been increasing every year since then whereas the RG3 isolates had been much more common before 2006 and numbers had fallen to very low levels since that time. As a consequence of the testing for ST64B_DT104_ we have been able to recognise these isolates as an emerging *S.* Typhimurium genotype possibly of Asian origin.

To further explore the status of RG9A members we selected from the years 2008 and 2009 thirty seven DT197 isolates with a variety of MLVA profiles for amplification of two contiguous DT104-specific SB26 sequences of 585 and 897 bp. All 37 isolates were positive for both sequences. Sequencing of both products for three isolates with different MLVA profiles showed that all three had identical sequences. The combined 1482 bp allele had 26 SNPs, a single nucleotide insertion and a single nucleotide deletion relative to the SB26 sequence in ST64B_DT104_ (GenBank Accession No. KC172925.1). The DT104-specific SB26 status of earlier DT197 isolates was also investigated (Text S1).

#### Variation in SB46 gene sequences

Testing for the SB46 gene common to both ST64B_DT64_ and ST64B_DT104_ showed that all selected members of RG3, RG4 and RG9 comprising mainly DT12a, DT101, DT179 and DT197 isolates were positive for SB46 even though they were not positive for other genes in either ST64B prophage. On sequencing of the 491 bp SB46 products it was found that they all had a sequence which had 9 SNPs compared with SB46 from ST64B_DT64_. The sequence of SB46 from ST64B_DT104_ has 3 SNPs (one located in one of the primers) compared with SB46 from ST64B_DT64_ and none of these is the same as the SNPs in the 9 SNP variant. When the SB46 product from a DT104 isolate belonging to RG8 was sequenced a mixture of the sequences from ST64B_DT104_ and the 9 SNP variant was found. BLASTing of the DT104 genome sequence with the 9 SNP SB46 sequence revealed a sequence with 100% identity to the 9 SNP variant located between coordinates 2832676 and 2833166. This lies within the location of Gifsy-1 on the DT104 genome [22]. BLASTing of the downloaded Gifsy-1 sequence from DT104 against the ST64B_DT64_ sequence showed extensive sharing of sequence between the two prophages including SB46 (detailed later). It was concluded that the isolates in RGs 3, 4 and 9 probably also had a Gifsy-1 sequence like that in DT104. Supporting evidence for this is presented in results for Gifsy-1 variants. The three closely related isolates with the apparent variant ST64B_DT104_ (see above) in RG7 (ATCC 13311, 05P41568972 DT1 and 09P84391818 DT41) were positive for the SB46 sequence with 9 SNPs. They were also shown later to have other features which are consistent with them having the Gifsy-1_DT104_ prophage.

The SB46 sequences in isolates which had tested positive for ST64B_DT64_ or ST64B_DT104_ were compared. SB46 for selected samples with each type of prophage was amplified and sequenced. Isolates with the ST64B_DT104_ prophage had the DT104 sequence with 2 SNPs (excluding one in the primer) relative to SB46 in ST64B_DT64_ (AY055382). Isolates with the ST64B_DT64_ prophage in RG2A (mainly DT141) and in all the Repeats Groups in Major Group 2 with the exception of those in RG14 (DT8, DT9 and DT64) had a sequence with one SNP relative to SB46 in ST64B_DT64_ (AY055382). This is the same sequence for SB46 in ST64B_DT64_ in the sequenced strains SL1344, DT2, D23580, ATCC 14028 and CVM23701. Isolates in RG14 had a mixed SB46 sequence consisting of the same 9 SNP variant seen in isolates presumed to have the Gifsy-1 sequence found in DT104 and either the same SB46 sequence seen in ST64B_DT64_ (AY055382) or the one SNP sequence seen in all of the other Repeats Groups in Major Group 2. As will be detailed later the Gifsy-1 sequence in RG14 isolates appears to be a hybrid between Gifsy-1 in DT104 and that in LT2. There were differences in distribution of ST64B SB46 sequence variants for the different phage types in RG14 (Text S2).

The SB46 gene sequence amplified from the presumed ST64B_DT104_ in isolates belonging to RG6B had 14 SNPs relative to the same sequence in ST64B_DT104_. The evidence indicates that the 14 SNP variant is located in the ST64B_DT104_ prophage rather than in the Gifsy-1 prophage and that the SB46 gene has been acquired from another serovar (Text S3).

### Comparison of Gifsy-1 sequences in sequenced strains

We made a comparison of the downloaded Gifsy-1 sequences from sequenced *S.* Typhimurium strains LT2, SL1344, DT104, DT2, D23580 and ATCC 14028. The sizes and genome coordinates for the six Gifsy-1 sequences are shown (Table 1). As a result of sequence comparisons (Text S4 and S5) we have been able to identify apparently unique sequences on Gifsy-1_DT104_ not shared by ST64B_DT64_ or ST64B_DT104_ or any of the other Gifsy-1 prophages, and on Gifsy-1_SL1344_ and Gifsy-1_DT2_ not shared by other Gifsy-1 prophages. These sequences were used to design primer pairs (Table 2) for PCR tests which could be used to determine what type of Gifsy-1 was present in *S.* Typhimurium isolates. There was almost no significant sequence on LT2 not found on one of the Gifsy-1 sequences or on Gifsy-2. However we located a sequence between 32761 and 35070 on Gifsy-1_LT2_ (NC_010392) which is not shared by Gifsy-2 or Gifsy-1_DT104_ but is shared by Gifsy-1_SL1344_ and Gifsy-1_DT2_. This was used to select primers for detection of possible Gifsy-1_LT2_ in isolates which were negative for Gifsy-1_SL1344_, Gifsy-1_DT2_ and Gifsy-1_DT104_-specific sequences.

**Table 1:** Sizes and genome coordinates of Gifsy-1 prophages for sequenced strains of *S.* Typhimurium.

Strain	Accession No.	Size (bp)	Coordinates
LT2 ATCC 43971	NC_003197	48491	2728552–2777042
DT104 NCTC 13348	NC_022569	49233	2796744–2845976
DT2 (Sanger)	NC_022544	50748	2719156–2769903
D23580	NC_016854	50767	2752645–2803411
ATCC 14028s	NC_016856	50772	2780105–2830876
SL1344	NC_016810	51187	2726269–2777455

**Table 2:** Primer sequences for phage loci, VNTR loci and CRISPR 1 and 2 in *Salmonella* serovar Typhimurium.

Primer	Sequence (5′-3′)	Product size bp	Target	Source
DT104 Gif1A-F	CACTCCAGACCTGCGCGACGATA	414	Gifsy-1_DT104_	NC_022569
DT104 Gif1A-R	CTGGTATGTGTGTCCCTCGCCG			
DT104 Gif1B-F	CGGCACCAGAACGGATCACGGCT	583	Gifsy-1_DT104_	NC_022569
DT104 Gif1B-R	GTAAAGCCAGACGTCAGGGAAACCG			
DT104 Gif1C-F	CCAGATCGTCAACTGTGTCATTCG	636	Gifsy-1_DT104_	NC_022569
DT104 Gif1C-R	TTGTATGGGACCTACGTTGCC			
DT104 Gif1D-F	CACAGAGCAACTGAATGCAATG	340	Gifsy-1_DT104_	NC_022569
DT104 Gif1D-R	GAGACGGATCTGGAACATCA			
DT104 Gif1E-F	CGGCGGAAGGCTGTAGAAACTA	644	Gifsy-1_DT104_	NC_022569
DT104 Gif1E-R	CGTCGTCGTATTGACTCTTGGC			
SL1344 Gif1A-F	CACGCCACTCACCGTTAGGC	413	Gifsy-1_SL1344_	NC_016810
SL1344 Gif1A-R	TCGCCTGTGTAATGACTACTGG			
SL1344 Gif1B-F	CCTCTTTGAACCCGGCTCGC	441	Gifsy-1_SL1344_	NC_016810
SL1344 Gif1B-R	GCAGGATGGCGCATTCCGTG			
SL1344 Gif1C-F	CGATTAGCCGTGGTAGCAGGG	495	Gifsy-1_SL1344_	NC_016810
SL1344 Gif1C-R	CTGAAGACCTAACTGCTGCCG			
SL1344 Gif1D.1-F	CTTCCAAGAGCAGCCCCTGTT	419	Gifsy-1_SL1344_	NC_016810
SL1344 Gif1D.1-R	CGATAGAGCTCAAGCAGGCGC			
SL1344 Gif1D.2-F	CCGGTTACCAGCCTTCATCAAG	500	Gifsy-1_SL1344_	NC_016810
SL1344 Gif1D.2-R	CAAGATCGACATCACCTGGAAGG			
SL1344 Gif1D.3-F	CCTTCCAGGTGATGTCGATCTTG	560	Gifsy-1_SL1344_	NC_016810
SL1344 Gif1D.3-R	CAAGCGTGTTGCCATCCTTCAC			
SL1344 Gif1E-F	GCGCTTACCACCTGTGTGGAA	446	Gifsy-1_SL1344_	NC_016810
SL1344 Gif1E-R	CATAATCCTTACCGCGGCCGG			
SL1344 Gif1F-F	CCGCGGCCTGCTGGTTAAG	431	Gifsy-1_SL1344_	NC_016810
SL1344 Gif1F-R	CGTACCAGTCCGGATCGCG			
DT2 Gif1A-F	CCCAAGTTAGAGATGCAAAACGCC	517	Gifsy-1_DT2_	NC_022544
DT2 Gif1A-R	CGTCGATCGACCATAATTTGGGC			
DT2 Gif1B-F	CACAACCCGGCCATGCTCAGG	421	Gifsy-1_DT2_	NC_022544
DT2 Gif1B-R	ATAAACCCCCTAAACCCCCCG			
DT2 Gif1C.1-F	GCAGGAACCACTCCTTGATAACG	566	Gifsy-1_DT2_	NC_022544
DT2 Gif1C.1-R	ATCCAGTTCCTTGAGACGAAGCG			
DT2 Gif1C.2-F	CGCCAAGAGGCAACTAAGTATCTG	386	Gifsy-1_DT2_	NC_022544
DT2 Gif1C.2-R	GTGAAAAATGCACATAATGACAAGCG			
DT2 Gif1 24bp Rpt-F	CGGGGGGGTTAGGGGGTTTAT	244	Gifsy-1_DT2_	NC_022544
DT2 Gif1 24bp Rpt-R	CGGTCAGCGAATGGTCATCTTTAC			
LT2 Gif1-F	GTGCTGTATCCAGTAGAAGCC	760	Gifsy-1_LT2_	NC_003197
LT2 Gif1-R	TCAGTCAGACACTACCATCGC			
CVM23701 Gif1A-F	GGCAGGAAGGGCTAATTCACGAGA	768	Gifsy-1_CVM23701_	ABAO01000067
CVM23701 Gif1A-R	CAATAGAGGCCGTGGCACTTGC			
CVM23701 Gif1B-F	GCAAGTGCCACGGCCTCTATTG	534	Gifsy-1_CVM23701_	ABAO01000067
CVM23701 Gif1B-R	GCGCATCACGACGAACGACG			
CVM23701 Gif1C-F	CGTCGTTCGTCGTGATGCGC	569	Gifsy-1_CVM23701_	ABAO01000067
CVM23701 Gif1C-R	CAGAACTTCGGTAGCGGATTTGC			
SPTR4-F	ACCTGGATAAATGGGCTTATTCCAAGC	474	Gifsy-1_CVM23701_	ABAO01000067
SPTR4-R	CATTCAGCCATTCGGGAACAGGAATAC			
Gifsy-3-F	GAGCTCAGCAACGTGTCGAAAGC	734	Gifsy-3_14028_	NC_016856
Gifsy-3-R	TTGGTCTGCGGGGACATCGCC			
DT104 SB52-F	GCGGCGATGAAGGAATTCGG	605	ST64B_DT104_	NC_022569
DT104 SB52-R	ACCAGACAAGACCACCAGCG			
DT104 SB53-F	CAGGAGGCGAATCAACATGCAA	538	ST64B_DT104_	NC_022569
DT104 SB53-R	CTCCAGCACTCACCCAAGATG			
DT104 SB26.1-F	CGGCGAAATTCTGTCTGGGTTATC	264	ST64B_DT104_	NC_022569
DT104 SB26.1-R1	CCTAAAGTGGTTCCCCTCTCAACT			
DT104 SB26.2-F	AAAGTTCAGACAATCAGCGTGG	388	ST64B_DT104_	NC_022569
DT104 SB26.1-R2	CCACGCTGATTGTCTGAACTTT	583		
DT104 SB26.3-F1	CCAGTATTTTCAGCGCAGTAG	553	ST64B_DT104_	NC_022569
DT104 SB26.2-R	CTACTGCGCTGAAAATACTGG			
DT104 SB26.3-F	CTCCAGGATGAGCTGCAGTAAC	444	ST64B_DT104_	NC_022569
DT104 SB26.3-R	CCAGGATCCTGACTCTTCTGTG			
DT104 SB33-F	GTCAGGCACTCATTAAAGGTTGCG	424	ST64B_DT104_	NC_022569
DT104 SB33-R	GGTTATCGATGCTTATCAGTCTGC			
DT104 SB3738-F	GGCTCGGTGGTATCCTTATAAG	359	ST64B_DT104_	NC_022569
DT104 SB3738-R	CTTTCAGAGATGGCTATTTCTTC			
DT104 SB49-F	GTCGAAAGCACCGCTTGGGTTTG	426	ST64B_DT104_	NC_022569
DT104 SB49-R	TAAACAGGCGTGCCATGAAGTCGG			
DT104 SB5051-F	GCATCGCGCCGACTGGCATATTT	381	ST64B_DT104_	NC_022569
DT104 SB5051-R	CCAACGGAACAGCACTGTATGTAAC			
STTR1-F	CAGCAGTACAACCGTCAGCAGGAT	770	45 bp TR	NC_003197
STTR1-R	GCCCCACCGTTAGCGCCCGATGTA			
STTR7-F	CGCGCAGCCGTTCTCACT	594	39/45/30 bp TR	NC_003197
STTR7-R	TGTTCCAGCGCAAAGGTATCTA			
STTR8-F	AATTAATTGCCGGATGGTGA	841	108/116 bp TR	NC_003197
STTR8-R	AGCGATTGCTGGCCTAGAT			
STTR11-F	ATCCAAGGGGTCGTTAGCTC	716	155 bp TR	NC_003197
STTR11-R	ACGTAGCCCCGTATCTGATG			
STTR12-F	GGATGGTGGTGTTATTGGCCGGT	762	184 bp TR	NC_003197
STTR12-R	CTGAAGGAGCACGCCTGGAAAGTG			
CDC ST3-F	GTTCTTCTGCAACGCAGGCA	193	12 bp TR	NC_003197
CDC ST3-R	GATGGCATGACGCTGCAACG			
CRSP1-F	CGAAGGCGGAAAAAACGTCCTG	1735	CRISPR1	NC_003197
CRSP1-R	GACGTATTCCGGTAGATCTGGATG			
CRSP1 74 bpSp-F	CGCGGGGAACACAATTAAAGCCGA	1273	CRISPR1 after 74 bp spacer	NC_003197
CRSP1 74 bpSp-R	TCGGCTTTAATTGTGTTCCCCGCG	486	CRISPR1 before 74 bp spacer	
CRSP2-F	GTASCWGCCATTACTGGTACACAG	2197	CRISPR2	NC_003197
CRSP2-R	CATAGCGATGCACGGATCACGC			
CRSP2-Sp13-F	ATTTCGCCTTCGGCACTGACGTCAC	1396	CRISPR2 after spacer 13	NC_003197
CRSP2-Sp13-R	GTGACGTCAGTGCCGAAGGCGAAAT	827	CRISPR2 before spacer 13	NC_003197
CRSP2-Sp24-F	CTAGGAGGCGTAATGAATACTACG	400	CRISPR2 after spacer 24	ABAO01000021
CRSP2-Sp24-R	CGTAGTATTCATTACGCCTCCTAG	1434	CRISPR2 before spacer 24	ABAO01000021

**Figure 2:**
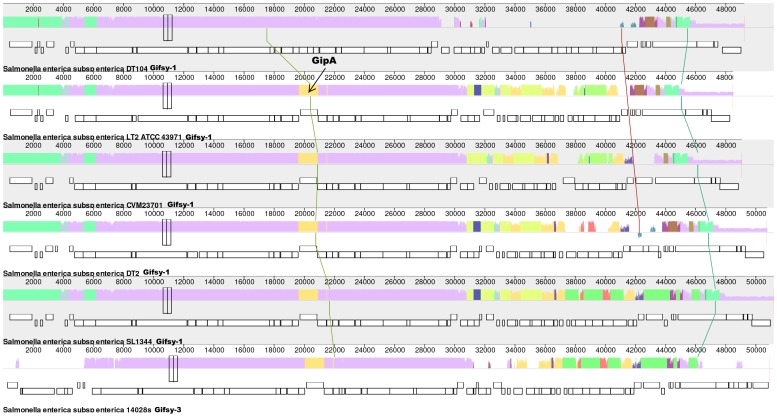
Comparison of Gifsy-1 sequences for *S.* Typhimurium strains LT2, DT104, SL1344, DT2 and *Salmonella* Serovar CVM23701 and Gifsy-3 sequence for *S.* Typhimurium ATCC 14028s by MAUVE (See also Text S5 for further explanation).

The unassembled sequenced strain *Salmonella* Serovar CVM23701 4,[5],12:i:- is located in RG12A (Figures S1 and S2) but BLASTing showed that it lacks the SL1344 and DT2 Gifsy-1 specific sequences. To locate the Gifsy-1 sequences in the contigs for CVM23701 4,[5],12:i:- the *Salmonella enterica* sequences in GenBank were BLASTed with the Gifsy-1_DT2_ sequence. This identified a sequence on the CVM23701 contig NZ_ABAO01000067 between coordinates 5611 and 9500 which is substituted for the Gifsy-1_DT2_ specific sequences. BLASTing of Salmonella sequences with this substitute sequence showed that the first 1274 bp sequence was shared only with *S.* Javiana GA_MM04042433 but the following 1464 to 3890 bp of the sequence showed over 96% alignment with Gifsy-1 and Gifsy-2 sequences of Typhimurium LT2 (as well as LT2-related strains) and with Gifsy-2 but not Gifsy-1 sequences for other Typhimurium strains. This part of the sequence was also found to contain a tandem repeat. In both Gifsy-1 and Gifsy-2 of LT2 it appears as three 22 bp repeats followed by an extended 39 bp repeat, two more 22 bp repeats and a 12 bp partial repeat. In the CVM23701 Gifsy-2 the tandem repeat has the same structure but in the CVM23701 Gifsy-1 it has the repeats structure 22×3, 39, 22×3, 39, 22×2, 12×1. In the D23580 Gifsy-2 the tandem repeat is like LT2 but lacks the 39 bp repeat. We designed primer pairs to be used for detection of Gifsy-1_CVM23701_-specific sequences in the first 1940 bases of the CVM23701 sequence as well as another pair from the later part of the sequence to amplify DNA shared by all Gifsy-2 sequences and the Gifsy-1 in LT2 (Table 2). In addition we chose primers to target the tandem repeat. Finally, a visual comparison of the five significantly different Gifsy-1 sequences together with Gifsy-3 from Typhimurium ATCC 14028S is shown (Figure 2).

### Testing of isolates for Sequences from Gifsy-1 variants

#### Distribution of Gifsy-1_DT104_-specific sequences

The three Gifsy-1_DT104_-specific sequences corresponded to positions 29105–29948 (a hypothetical protein in Gifsy-1 phages of several other Serovars), 38294–39171 (Replication protein O in several others Serovars) and 31778–34537 (within the *artAB* gene complex Accession No. AB104436.1). The results of tests for the three Gifsy-1_DT104_-specific sequences in representative isolates from the panel of 214 well-characterised isolates (Figures S1 and S2) showed that isolates in RGs 3, 4, 8, 9A and 9B which are all in Major Group 1 had all three sequences and also lacked the GipA sequence. These RGs included DT179, DT101, DT12a, DT104 and most of the DT197 isolates. It was concluded that all the isolates in these RGs probably have the Gifsy-1_DT104_ prophage. It was noted that all the tested DT101 and DT12a isolates had the Gifsy-1_DT104_ sequences even though each phage type consisted of isolates with distinctly different genotypes (Figures S1 and S2). Isolates in RG14 in Major Group 2 consisting of DT8, DT9 and DT64 isolates had the GipA sequence and two of the Gifsy-1_DT104_ sequences but lacked the sequence corresponding to the 29105–29948 insert in Gifsy-1_DT104_. These isolates appear to have a hybrid form of Gifsy-1 in which the first part of the genome to beyond the insertion point for the insert is like that seen in LT2, DT2 or SL1344 and the remainder is like that seen in Gifsy-1_DT104_. We have designated this prophage Gifsy-1_DT64_. Sequencing of a selection of PCR products for all three Gifsy-1_DT104_-specific sequences from each of the RGs showed no difference from the sequences in DT104 except for isolate 06P23331584 in RG3 (Figure S1) (Text S6).

#### Distribution of Gifsy-1_SL1344_-specific sequences

The results of tests for the six Gifsy-1_SL1344_-specific sequences in representative isolates from the panel of 214 well-characterised isolates (Figures S1 and S2) showed that isolates in RG10A and RG10B (mostly DT44, DT29 and DT35 isolates, along with some DT120, DT186, U302 isolates and including strain SL1344) in Major Group 2 had all six sequences as well as the GipA sequence and therefore were presumed to have the Gifsy-1_SL1344_ prophage. The DT126 isolates in RG11A and in RG11B (also in Major Group 2) had all six Gifsy-1_SL1344_ sequences as well as the GipA sequence but subsequent testing showed that they lacked the Gifsy-1_LT2_ sequence also found in Gifsy-1_SL1344_ and Gifsy-1_DT2_ but not in Gifsy-1_DT104_. This result was supported by the same result in a further 19 DT126 isolates. As this indicates a significantly different sequence from Gifsy-1_SL1344_ we have designated this prophage Gifsy-1_DT126_. In Major Group 1 RG2 isolates which were mostly phage typed as DT141 and variants of DT141 but not any of the DT4 isolates (including LT2) had all six of the Gifsy-1_SL1344_ sequences as well as GipA and the Gifsy-1_LT2_ sequence also found in Gifsy-1_SL1344_ and Gifsy-1_DT2_ but not in Gifsy-1_DT104_. They were presumed to have the Gifsy-1_SL1344_ prophage. All the DT141 and DT141 variants which were tested for CRISPR sequences had the RG2A-type CRISPR 2 sequences.

#### Distribution of Gifsy-1_DT2_-specific sequences

The results of tests for the three Gifsy-1_DT2_-specific sequences in representative isolates from the panel of 214 well-characterised isolates (Figures S1 and S2) showed that isolates in RG1 (mainly DT170 and DT12 but with some DT186 and DT193 isolates) in Major Group 1 had all three sequences as well as GipA and were presumed to have the Gifsy-1_DT2_ prophage. Selected isolates in all the divisions of RG12 and RG13 in Major Group 2 as well as 06P75527334 in RG10A (Figure S2) also had all three sequences as well as GipA and were presumed to have the Gifsy-1_DT2_ prophage. RG12 and RG13 were populated with mostly DT135, DT135a* and DT170 isolates with smaller numbers of U307, DT102, DT6 var 1, U290 and DT3 isolates. Significantly, isolate 06P75527334 was typed as DT135a which was different from the other phage types in RG10A. Its location there was determined solely by its CRISPR 2 profile since its CRISPR 1 profile placed it in RG12D (Figure S1). Isolate 06P75527334 is one of a number of hybrid isolates in the panel in which the CRISPR 1 and 2 profiles belong to different Repeats Groups. The sequenced strains ATCC 14028 and D23580 have been placed in RG12A and RG12B respectively and have the Gifsy-1_DT2_ prophage but DT2 itself has not been assigned to a Repeats Group because its CRISPR profile does not conform to any of the recognised Repeats Groups. Isolate 08P88211137 in RG12A was found to have all three Gifsy-1_DT2_-specific sequences. This strain had previously been found to have all six Gifsy-1_SL1344_-specific sequences. It was subsequently shown that these sequences are located on the Gifsy-3 prophage present in this strain (see below). One isolate assigned to RG2B had all three Gifsy-1_DT2_-specific sequences and another also in RG2B had just one Gifsy-1_DT2_-specific sequence but as will be shown later most RG2B isolates have the Gifsy-1_LT2_ variant.

#### Identification of Gifsy-1sequences specific to CVM23701 in selected isolates

Using the primer pairs derived from CVM23701 to test a limited range of isolates we showed that the CVM23701-specific sequences and the larger tandem repeat sequence found in this strain were present in only one local isolate (07P69570074) from 2007 which had a RDNC phage typing result. This isolate significantly lacks SL1344 and DT2 Gifsy-1 specific sequences and is diphasic not monophasic. This isolate has the same MLVA and CRISPR profiles as CVM23701 and appears to have the same Gifsy-1 prophage as CVM23701. We have therefore designated this prophage Gifsy-1_CVM23701_. We obtained DNA from a strain of DT191a isolated in the UK in 2008 and associated with an outbreak of human salmonellosis traced to handling of feeder mice imported from the USA [24], [25]. Like CVM23701 this strain is monophasic. It also lacks the SL1344 and DT2 Gifsy-1 specific sequences but has the two CVM23701 Gifsy-1-specific sequences. However it lacks the larger tandem repeat sequence found in CVM23701 and local isolate 07P69570074. All isolates tested for the tandem repeat located on Gifsy-2 (and Gifsy-1 in LT2) had the same size allele and not the shorter allele found in D23580.

#### Identification of isolates likely to have the Gifsy-1_LT2_ variant

Since there were no unique sequences found in Gifsy-1_LT2_ we applied a PCR test for amplification of a Gifsy-1_LT2_ sequence found also in Gifsy-1_SL1344_ and Gifsy-1_DT2_ but not in Gifsy-2 or Gifsy-1_DT104_ as a proxy test for the possible presence of Gifsy-1_LT2_. Isolates negative for Gifsy-1_DT104_, Gifsy-1_SL1344_ and Gifsy-1_DT2_ -specific sequences but positive for GipA and STTR6 were tested. Isolates in RG2 (DT4, DT193) which had not tested positive to one of the other Gifsy-1 types as well as additional DT4 isolates were all positive for the Gifsy-1_LT2_ sequence. They most likely have Gifsy-1_LT2_ because LT2 is DT4 and belongs to this Repeats Group. All tested isolates with the 171-x-x-0-489 MLVA in RG6B were positive as were all DT13 isolates, a related DT41 isolate and an anomalous DT197 isolate in RG11A as well as a DT160 isolate and a closely related untypable isolate, the only members of RG15. Sequencing of the Gifsy-1_LT2_ sequence from a DT4 and a DT193 (RG2) isolate showed 100% identity with the sequence in LT2. However, sequencing of the same product from four isolates with the 171-x-x-0-489 MLVA belonging to RG6B as well as the untypable strain 08P66217254 (in RG1 in Figure S1 and RG4A in Figure S2) and 07P25565432 typed as DT197 (but with a unique STTR1 allele and CRISPR 1 and 2 sequences most closely related to those of DT126 isolates in RG11A) showed alleles with a single SNP at base 643.

As a validation test we tested a range of isolates positive for Gifsy-1_DT104_, Gifsy-1_DT64_, Gifsy-1_SL1344_ or Gifsy-1_DT2_ sequences for the presence of the Gifsy-1_LT2_ sequence. All of the isolates positive for Gifsy-1_DT104_ or Gifsy-1_DT64_ were negative for the Gifsy-1_LT2_ sequence. All of the isolates from various Repeats Groups which were positive for Gifsy-1_SL1344_ or Gifsy-1_DT2_ were positive for the Gifsy-1_LT2_ sequence except for DT126 and DT126 var 4 isolates in RG11A and B, which had previously tested positive for all of the Gifsy-1_SL1344_ sequences and yet tested negative for the Gifsy-1_LT2_ sequence. As previously indicated we have designated this prophage Gifsy-1_DT126_.

### Distribution of Gifsy-3_14028_ sequence

Isolate 08P88211137 in RG12A, phage typed as DT135, was positive for all six Gifsy-1_SL1344_ sequences even though other DT135 isolates were negative for all six. It was found that this isolate was also positive for Gifsy-1_DT2_-specific sequences as were other DT135 isolates. This isolate is one of a small group of isolates placed in RG12A of Major Group 2 in Figures S1 and S2 because the CRISPR 2 profile was the same as for strains CVM23701 4,[5],12;i;- and ATCC 14028s although the CRISPR 1 profiles were the same as other DT135 and DT102 isolates in RG12B in Figure S1. BLASTing of the ATCC 14028s sequence with the Gifsy-1_SL1344_ sequence showed that there was an alignment at 99% identity but in reverse order for the Gifsy-1_SL1344_ sequence 36491 to 46388 (Figure 2) with ATCC 14028s sequence (NC_016856) at coordinates 1289242 to 1299140. This included the whole region where the six Gifsy-1_SL1344_ sequences are located. This was known to be the approximate location of the Gifsy-3 prophage [21]. Following this discovery it was noted that the MLVA profile of isolate 08P88211137 (DT135), 162-300-318-357-523, was very similar to that for ATCC 14028s, 162-306-318-357-523. We therefore explored our *S.* Typhimurium isolate database from 2006 onwards for MLVA profiles the same as those for 08P88211137 or ATCC 14028. We found seven isolates with the same MLVA as 08P88211137 including two from kangaroo meat and one with the same MLVA as ATCC 14028s. All were typed as DT135 except one of the isolates with the 08P88211137 MLVA which was RDNC and the two kangaroo isolates one typed as DT12 and the other as RDNC. On testing for Gifsy-1 sequences all were positive for both Gifsy-1_SL1344_ and Gifsy-1_DT2_ sequences. By alignment of the downloaded 50884 bp Gifsy-3_14028_ sequence (ATCC 14028s genome coordinates 1284562 and 1335445) with the various Gifsy-1 sequences and the downloaded Gifsy-2 sequence we located a sequence at 46191 to 47760 on Gifsy-3_14028_ (Figure 2) with genome coordinates 1330752 and 1332321 which appeared to be specific to Gifsy-3_14028_. We chose primers for a 734 bp internal sequence and tested all of the isolates with MLVA same or similar to ATCC 14028s and a representative sample of 33 isolates positive only for the Gifsy-1_SL1344_ -specific sequences. Only the seven isolates previously positive for both Gifsy-1_SL1344_ and Gifsy-1_DT2_ sequences were positive for the Gifsy-3_14028_ sequence. PCR product from two of the positive isolates was sequenced and shown to have 100% identity with the Gifsy-3_14028_ sequence.

### Correlation of Prophage Profiles with Repeats Groups and Phage Types

On the basis of the two ST64B prophages including two variants of ST64B_DT104_, seven Gifsy-1 prophages, Gifsy-3 prophage and a variant of SB26 gene from ST64B_DT104_ we were able to distribute almost all *S.* Typhimurium isolates into sixteen prophage profiles (Table 3). Some profiles were exclusive to one Repeats Group while others were common to two or several Repeats Groups. RG11A contained two profiles and RG12A contained three. There was a high degree of correlation between prophage profiles and phage types with many phage types falling completely or almost completely within a single prophage profile. Some phage types, notably DT120, DT170, DT186, DT193, DT6 var 1, DT1 and U302, were represented in two or more prophage profiles usually consistent with the Repeats Group to which they had been assigned. Thus DT170 isolates fell almost entirely into two distinct Repeats Groups (1 and 13A) which correlated completely with prophage profiles 6 and 8 respectively. Both profiles had Gifsy-1_DT2_ but profile 6 isolates had ST64B_DT104_ and profile 8 isolates had ST64B_DT64_.

**Table 3:** Distribution of Gifsy-3, variants of Gifsy-1 and ST64B, and the SB26 gene of ST64B_DT104_ among Repeats Groups and phage types of *Salmonella* serovar Typhimurium.

				ST64B			Gifsy-1							Gifsy-3
Prophage profile	Repeats Group	Major Group	Principal Phage Types or Strains	DT64	DT104	SB26_DT104_	LT2	DT104	DT64	CVM23701	SL1344	DT126	DT2	
1	2B^1^	1	4, 193, **LT2** (RG2A)	−	−	−	+	−	−	−	−	−	−	−
2	6B	1	193, 120, 195, Untyp (171-x-x-0-489 MLVA)	−	+^3^	+	+	−	−	−	−	−	−	−
3 or 4	2A	1	141, 141 var 1&2	+/−	−	−	−	−	−	−	+	−	−	−
4	10A, B	2	44, 29, 35, U302, 186, **SL1344**	+	−	−	−	−	−	−	+	−	−	−
5	11A, B	2	126, 126 var 4	+	−	−	−	−	−	−	−	+	−	−
6	1	1	170, 12, 186, 193	−	+	+	−	−	−	−	−	−	+	−
7	12A	2	135 with ATCC 14028-like MLVA, **ATCC 14028**	+	−	−	−	−	−	−	−	−	+	+
8	12A, B, C, D	2	135, 135a, 102, U307, **D23580**	+	−	−	−	−	−	−	−	−	+	−
8	13A, B, C	2	135, 135a, 170, U302, 6 var 1	+	−	−	−	−	−	−	−	−	+	−
9	11A	2	13	+	−	−	+	−	−	−	−	−	−	−
9	15	2	160	+	−	−	+	−	−	−	−	−	−	−
10	14A, B	2	8, 9, 64	+	−	−	−	−	+	−	−	−	−	−
11	12A	2	**CVM23701**, **UK-1, str 08-1736**, 07P69570074 (RDNC)	+	−	−	−	−	−	+	−	−	−	−
12	3	1	101, 12a	−	−	−	−	+	−	−	−	−	−	−
12	4A, B	1	179, 101, 120, 104L	−	−	−	−	+	−	−	−	−	−	−
12	9B	1	12a	−	−	−	−	+	−	−	−	−	−	−
13	7	1	1, 41, ATCC 13311	−	+^4^	+	−	+	−	−	−	−	−	−
14	8	1	104, 104L, 12L, **DT104**	−	+	+	−	+	−	−	−	−	−	−
15	9A	1	197, 43, 6, 6 var 1	−	−	+^5^	−	+	−	−	−	−	−	−
16	5A, B	1	120, 193	−	−	−	−	−	−	−	−	−	−	−
16	6A^2^	1	22, 21	−	−	−	−	−	−	−	−	−	−	−

1 Rare isolates have Gifsy-1_DT2_ instead of Gifsy-1_LT2_.

2 Rare isolates may have ST64B_DT104_ or Gifsy-1_DT104_.

3 and ^4^ are sequence variants of ST64B_DT104_.

5 A sequence variant of SB26_DT104_.

## Discussion

Previous studies have reported significant sequence differences for ST64B and Gifsy-1 prophages from different strains of *S.* Typhimurium but they have by and large not investigated these differences further and they have not mapped the distribution of these variants among isolates with a diverse range of genotypes and phage types. By comparison of the available sequenced prophages we have shown that there are two significant variants of ST64B: the prototype from DT64, which is almost the same in strains SL1344, DT2, D23580, ATCC 14028s and CVM23701 4,[5],12:i:-; and the one in DT104. We have shown likewise that there are five significant variants of Gifsy-1 amongst sequenced strains, one in LT2, one in DT104, one in SL1344, one in CVM23701 4,[5],12:i:- and one shared by DT2, D23580 and ATCC 14028s. By comparison of the variants of each prophage we have identified sequences which appear unique to a particular prophage variant or, in the case of Gifsy-1_LT2_, shared by a number of variants. Our investigations have led to the rediscovery that significant gene sequences are shared between ST64B and Gifsy-1 prophage from DT104 and made the further rediscovery that a large segment of Gifsy-1 from SL1344 is shared by Gifsy-3 from ATCC 14028s. We have used the techniques of PCR and sequencing of PCR products to test for the presence of these ‘unique’ sequences in *S.* Typhimurium isolates, from a large collection of local isolates, chosen to represent most of the phage types encountered locally and most of the genotypes we have identified by VNTR and CRISPR analysis over the period 2000 to 2012. As a result of extensive testing we have identified two new variants of Gifsy-1, one in DT126 isolates and one in DT8/9/64 isolates, as well as two variants of ST64B_DT104_. We have now found that the sequenced *S.* Typhimurium strains UK-1 (NC_016863) [26] and 08-1736 (NC_021820) contain a Gifsy-1 prophage which is very closely related to the one in CVM23701. It has the same sequence which we have shown to be unique to CVM23701 and a local strain as well as the extended 22 bp VNTR also only found in those two strains.

To identify a particular prophage variant in an isolate we have made the assumption that an isolate which has all of the sequences thought to be unique to a particular variant as well as all of the tested sequences shared by that variant with other variants is likely to have a prophage which is the same as or closely related to that variant. Support for this contention comes from the *S.* Typhimurium whole genome sequences deposited as Whole Genome Shotgun projects at DDBJ/EMBL/GenBank under the accessions AMDX, AMDY, AMEA, AMEB, AMEC, AMED, AMEE, AMEF, AMEG, and AMEH. All the isolates are strains of DT135a and belong to the RG13A genotype. The Gifsy-1 sequences on most of these strains have been located and found to be almost identical to the Gifsy-1_DT2_ variant sequence with the notable exception of a 24 bp VNTR located at 2172456 to 2172555 on the assembled DT2 genome (Sanger) (Text S7). The sequence for *S.* Typhimurium strain TN061786 under accession AERV01000000.1 has a Gifsy-1 sequence almost identical to the Gifsy-1_DT2_ variant sequence as well as the same 24 bp VNTR. TN061786 is a hybrid strain which like local isolate 08P66217254 belongs to RG1 in Figure S1 and RG4 in Figure S2. Further support from another angle comes from the Southern hybridisation analyses of Tucker and Heuzenroeder [17] (Text S8).

We have been able to map the distribution of Gifsy-3 and variants of ST64B and Gifsy-1 prophages among local isolates. We have found a very high degree of correlation between the VNTR/CRISPR genotype which we have termed Repeats Group and the prophage profile based on ST64B and Gifsy-1 variants (Table 3). We believe that this correlation lends support to the concept of VNTR/CRISPR genotypes or Repeats Groups in *S.* Typhimurium. There is a very similar system of genotyping applied to isolates of *Mycobacterium* [27], [28], [29]. Some Repeats Groups such as RGs 1, 6B, 8 and 14 have all or nearly all members with the same unique prophage profile. Others may share their prophage profile with one or more Repeats Groups while some Repeats Groups are divided into two or more different profiles. Further differentiation can be obtained by including Gifsy-3 for some members of RG12A and the SB26_DT104_ variant found in DTs 197, 43 and 6 in RG9A. Results of phage gene profiling [19] show that more differentiation can be obtained by including markers from other phage groups such as P22- and P2-like phages.

Some phage types such as DTs 4, 8, 9, 12a, 22, 44, 64, 101, 179 and 197 correlate totally or almost so with a single prophage profile even for DTs 12a and 101 which are divided into two different Repeats Groups. Others such as DTs 170 and 6 var 1 correlate with two distinct prophage profiles. Thus isolates with one prophage profile have a genotype and a Repeats Group which are different from isolates with the other prophage profile. Still other phage types such as DTs 120, 186 and 193 have quite a diverse range of genotypes and prophage profiles. This is most likely because these phage types show very few reactions or none (DT193) with the Anderson typing phage panel.

We have tried to assess how much influence the different variants of ST64B and Gifsy-1 may have on phage typing. In the case of DTs 4 and 141 there is evidence that the correlation of DT4 with the Gifsy-1_LT2_ variant and of DT141 with the Gifsy-1_SL1344_ variant may be related to the difference in phage typing pattern. It may be supposed that since most of the Anderson typing phages are P22 phages [30] it would be likely that phage type would be more greatly influenced by the type of P22 phage in an isolate. It is known that conversion of DT9 to DT64 and of DT135 to DT16 is caused by lysogenisation with ST64T, one of the P22 phages [3]. However DT4 and DT141 lack P22 phages by phage gene profiling [19] so it seems possible that the different Gifsy-1 variants may be contributing to the observed phage type difference. Arguing against this is the example of isolates with the phage type 6 var 1. Some have the combination of ST64B_DT64_ with Gifsy-1_DT2_ while others have the combination of the SB26_DT104_ variant with Gifsy-1_DT104_ but no ST64B prophage. There appears to be no P22 phage by phage gene profiling so the phage type appears to be unaffected by the difference in prophage profiles. Perhaps some Gifsy-1 variants influence phage typing patterns but others do not. The example of DT170 where isolates of two distinct genotypes with prophage profiles which have different ST64B variants but the same Gifsy-1 and apparently the same P22 prophage [19] produce the same phage typing pattern appears to indicate that the difference in ST64B phage sequence does not affect the phage type. Some DT141 isolates have ST64B_DT64_ while others lack the prophage and yet the phage typing result is not affected. However, Tucker and Heuzenroeder [17] observed changes in phage type following introduction of the *imm-C*-like genes from ST64B_DT64_ into isolates.

We have divided all *S.* Typhimurium isolates into two groups termed Major Groups 1 and 2. The division is based mainly on the different alleles of STTR3, STTR7 and STTR9 (Text S9). We have noted that isolates in Major Group 2 practically always have the SB26 gene from ST64B_DT64_ while isolates in Major Group 1 always lack the gene with the exception of a minority of members of RG2A, mostly DT141. We also note that the phage profiling results indicate that ST64B prophages seem only to occur when a Gifsy-1 prophage is present in an isolate. Thus the marked difference in occurrence of ST64B_DT64_ between the two Major Groups is possibly related to the type of Gifsy-1 variant or combination of Gifsy-1 variant and ST64B variant present in an isolate. All isolates in Major Group 2 have ST64B_DT64_ combined with one of six Gifsy-1 variants. In contrast isolates in Major Group 1 may have both ST64B and Gifsy-1 prophages, Gifsy-1 but no ST64B or lack both ST64B and Gifsy-1 prophages. Four of the Repeats Groups in Major Group 1 have ST64B_DT104_ or one of its variants in combination with one of three Gifsy-1 variants and RG2A has some DT141 isolates with ST64B_DT64_ combined with Gifsy-1_SL1344_. The latter combination is the only one which also occurs in Major Group 2 isolates. Gifsy-1_DT2_ occurs in both Major Groups but in Major Group 1 it is combined with ST64B_DT104_ while in Major Group 2 it is combined with ST64B_DT64_. Apparently Gifsy-1_LT2_ occurs in both Major Groups but only in RG15 isolates (DT160 and related Untypable isolate) and DT13 isolates belonging to RG11A is it accompanied by ST64B_DT64_. Since it is known that the phage type of a strain can be altered by the introduction of a new phage lysogen it is not surprising that there is a degree of correlation between phage type and prophage profile. However it is difficult to explain the even higher level of correlation between the repeats profile and prophage profile even though the STTR6 VNTR is located on Gifsy-1, and CRISPR sequences are known to carry spacer sequences which can be derived from prophages and are known to confer immunity from infection with phages which carry the same sequences as in the spacers [31]. The composition of the Repeats Groups depends as much on the combinations of VNTR alleles as it does on the CRISPR profiles.

It is possible that superinfection exclusion systems controlled by residing prophages is driving these divisions. Gifsy-1_DT104_ and probably Gifsy-1_DT64_, from the results of Tucker and Heuzenroeder [17], have the same *immC* region (SB38, 39 and 40 in the ST64B_DT64_ genome) as ST64B_DT64_ and ST64B_DT104_ but the other Gifsy-1 variants do not. Consequently it may be expected that any isolate with Gifsy-1_DT104_ or Gifsy-1_DT64_ may be immune to infection by an ST64B phage. There is partial evidence for this in the table of prophage profiles. Thus four out of six Repeats Groups in Major Group 1 with Gifsy-1_DT104_ do not have any ST64B prophage. However, in the case of isolates in RG8, mainly DT104, dual lysogeny with ST64B_DT104_ and Gifsy-1_DT104_ is permitted. It should be noted that co-existence of two prophages with the same immunity module occurs in LT2 with Gifsy-1 and Gifsy-2 [15]. It is presumed that this is the result of an intrachromosomal recombination/conversion event [13]. This may be the case also for ATCC 13311 and related members of RG7. However the ST64B_DT104_ variant in these isolates may not have the ST64B *immC* region because it has the two ST64B_DT104_-specific sequences before the *immC* region but not the ones after it. All the isolates in Major Group 2 have ST64B_DT64_ even in RG14 isolates which have Gifsy-1_DT64_ which possibly has the same immunity module as ST64B_DT64_. RG14 isolates may therefore represent another example of recombination producing lysogeny with two prophages with the same immunity module. Even allowing for exceptional recombination events it is still possible that superinfection exclusion systems are responsible for maintaining clones and hence for the strong correlations we see between genotype, phage type and prophage profiles. For example, it is curious that members of RG1 in Major Group 1 with Gifsy-1_DT2_ have ST64B_DT104_ while all the isolates in Major Group 2 with Gifsy-1_DT2_ have ST64B_DT64_. Then again, members of RG2B in Major Group 1 with Gifsy-1_SL1344_ may or may not have ST64B_DT64_ while all members of RG10 in Major Group 2 with Gifsy-1_SL1344_ have ST64B_DT64_. It may be that certain combinations of prophage are difficult to disrupt and so clones arise because they are protected from the genetic changes resulting from invading phages. These clones survive if they have selective advantage but are gradually replaced as new strains with a more advantageous mix of prophages and other mobile elements arise. This is consistent with the conclusion of Figueroa-Bossi et al 2001 [12] that lysogenic conversion is a major mechanism driving the evolution of *Salmonella* bacteria. There are also P22 and P2-like prophages which if present are likely to contribute to the maintenance of clones.

We have identified an emerging *S.* Typhimurium genotype with phage types DT193, DT120, DT195, U302, RDNC and Untypable with a 171-x-x-0-489 MLVA profile and multiresistant antibiotic profiles. Possibly the same genotype has been reported in the United Kingdom [29] where DT120 isolates had similar or same MLVA and antibiotic resistance profiles as seen here. A study of *Salmonella enterica* Serovar 4,[5],12:i:- monophasic variants of *S.* Typhimurium isolated in Germany from pigs, pork and humans in 2006 and 2007 [32] showed that the most common phage types were DT193 and DT120, most MLVA profiles were 171-x-x-0-489 and most were multiresistant with the most common resistance profile the same as reported in our isolates and in the UK. It would be interesting to compare the prophage profiles for the isolates from both countries to see if they are the same as for the emerging genotypes we are seeing in Australia. It should be noted that strain CVM23701 4,[5],12:i:- included in our table of isolates is a different genotype from nearly all of the isolates in the German study and it has a different prophage profile from our isolates with the 171-x-x-0-489 MLVA (see [33]).

We have made the interesting discovery that isolates in RG9A with phage type DT197, DT6 and DT43 have a sequence variant of the ST64B_DT104_ gene SB26_DT104_ but have no other tested ST64B_DT104_ sequences. Isolates with phage type DT12a (RG9B) lack the SB26_DT104_ variant. They are placed in RG9B because the MLVA profiles are closely related to those of DTs 197, 6 and 43, the CRISPR 1 profiles are identical to these other phage types and the CRISPR 2 profiles are related, but DT12a isolates have significantly more spacers than DTs 197, 6 and 43 isolates. It seems probable that SB26_DT104_ is located on another prophage. It also seems likely that DTs 197, 6 and 43 have arisen from DT12a (RG9B) by acquisition of further prophage or prophages and a simultaneous deletion of CRISPR 2 spacers. We note from our phage gene profiling results [19] that DT12a isolates have markers for a P22 prophage not present in DTs 197, 6 or 43 isolates but DTs 197, 6 and 43 isolates have the *SopE* marker from SopEΦ, a P2-like prophage, not present in DT12a. However testing (not shown) indicates that the SopEΦ insertion point in DT197 isolates is vacant and the *SopE* product from DT197 isolates has 5 SNPs relative to the *SopE* sequence in SopEΦ. Therefore the *SopE* gene variant in DT197 must have another location perhaps in another phage. Sequencing of DT197 and DT12a strains would be desirable to elucidate their relationship.

The detection of seven isolates among thousands with the rare Gifsy-3 prophage by the similarity of their MLVA profiles to that of ATCC 14028 is strong evidence that MLVA profiles have a significant probability of predicting phylogenetic relationships between strains. It also indicates that clones of related strains are relatively constant over time since ATCC 14028 was isolated in 1960 [21]. Clonal constancy is also demonstrated by the unexpected discovery of relatedness between ATCC 13311 and two local isolates from 2005 and 2009 which have almost the same MLVA as ATCC 13311 and the same unique ST64B_DT104_ variant as well as Gifsy-1_DT104_. ATCC 13311 was isolated in 1911 probably in the Netherlands [34]. 05P41568972 DT1 was isolated in 2005 from an adult male who is the neighbour in a suburb of Brisbane of the adult male from whom 09P84391818 DT41 was isolated in 2009. The latter patient is thought to have acquired his *Salmonella* infection in Vietnam.

The two members of RG15 are: MDU 2009 16 which was isolated in Tasmania and typed as DT160 which is the same as the phage type associated with infection in birds and humans in New Zealand since 2001 [35]; and 05P08494937 which was isolated in 2005 from a Queensland patient and was phage Untypable. The MLVA profiles for the two isolates are closely related. Both have unique STTR3, STTR7 and STTR8 alleles and an STTR12 allele rarely seen in isolates other than DT126 and DT13 isolates. Both CRISPR 1 and 2 profiles are unique. The CRISPR 1 profiles are the same for the two isolates but the CRISPR 2 profiles are significantly different although clearly related. Both isolates have ST64B_DT64_ and probably Gifsy-1_LT2_ as far as our tests can reveal. This prophage combination is only shared with DT13 isolates in RG11A. Clearly there is potential for this type of genotyping to discover more diversity among *S.* Typhimurium isolates from other animal and environmental niches.

## Conclusions

As a result of a survey of characterised *S.* Typhimurium isolates seeking specific sequences of prophage variants we have discovered at least two additional variants of Gifsy-1, one in isolates of DTs 8, 9 and 64 which appears to be a hybrid of non-DT104 Gifsy-1 and Gifsy-1_DT104_, another which appears to be specific to DT126 isolates and recognised a third in *Salmonella* CVM23701 (GenBank ABAO00000000.1) and a local isolate. We have also made the rediscovery that Gifsy-1 in SL1344 shares specific sequences with Gifsy-3 in ATCC 14028. A search for isolates which have an MLVA profile the same as or similar to that of ATCC 14028 has allowed us to identify, among several thousands, a few rare isolates which harbour this prophage. On the basis of two ST64B prophages including two variants of ST64B_DT104_, seven Gifsy-1 prophages, Gifsy-3 prophage and a variant of the substituted SB26 gene from ST64B_DT104_ we were able to divide almost all *S.* Typhimurium isolates into sixteen profile groups. Each of these groups is specific, or largely so, to a range of phage types. In addition we find that each prophage profile is specific to one or a number of genotypes (Repeats Groups) as determined by the combination of VNTR and CRISPR typing.

## Materials and Methods

### Bacterial isolates and DNA preparation

The isolates of *S.* Typhimurium used in this study were nearly all obtained from specimens collected in Queensland and northern New South Wales. They were mostly derived from cases of human infection. The majority of them were locally-acquired but significant numbers could have been acquired overseas. They were recruited mainly from the approximately 2,500 isolates routinely typed by MLVA at the Public Health Microbiology Laboratory in the period 2006 to 2009 and from a similar number of retrospectively MLVA-typed isolates from the 1999–2005 period. A panel of 214 isolates which had been well characterised and assembled into related groups termed Repeats Groups (RGs) according to their VNTR and CRISPR profiles was tested most intensively (Figures S1 and S2). When correlations between phage gene test results and RG or other groups of isolates were observed, further isolates with same or similar MLVA profiles or phage type were selected to test the correlations more rigorously. In addition, a culture of *S.* Typhimurium ATCC 13311 was obtained from the American Type Culture Collection. DNA was prepared by taking one colony from an overnight culture into 400 µl of TE buffer and boiling for 8–10 minutes.

### Comparison of ST64B, Gifsy-1 and Gifsy-3 Sequences

The sequence for ST64B_DT64_ Accession No. AY055382 and the gene map were obtained from GenBank (http://www.ncbi.nlm.nih.gov/genbank/). The whole genome sequences for *S.* Typhimurium DT2 and DT104 were downloaded from the Sanger website (http://www.sanger.ac.uk/Projects/Salmonella). The Align program in NCBI website was used to align the ST64B_DT64_ sequence with each of the DT2 and DT104 sequences to locate the boundaries of the ST64B_DT64_ prophage in each genome. The ST64B sequences in each strain were then selected out. The integration point for the ST64B_DT64_ prophage is located at 21042 which is between SB27 and SB28 around the midpoint of the ST64B_DT64_ genome. The comparison of the DT104 sequence was therefore more conveniently done against the sequence from DT2 which was very close to the ST64B_DT64_ sequence. This was done using BLASTn from the NCBI Align program. Maps showing the aligned and non-aligned regions were constructed. The gene map from ST64B_DT64_ was used to determine the positions of the genes and intergenic regions for the DT2 sequence so that the corresponding positions on the DT104 sequence could be located. The non-aligned regions and areas of relatively low alignment were located and were extracted from each of the DT2 and DT104 sequences. A visual comparison of the ST64B sequences for DT2 and DT104 was made using MAUVE [20].

A similar process was followed for Gifsy-1. Additional whole genome sequences for ATCC 14028 (GenBank Accession No. NC_016856) and SL1344 and D23580 (Sanger site) were downloaded. The Gifsy-1_LT2_ sequence Accession No. NC_010392.1 was BLASTed against the DT104 whole genome sequence to locate the 5′ boundary and the following 50,000 base sequence was downloaded. The LT2 and DT104 Gifsy-1 sequences were aligned and a map was constructed to identify the aligned and non-aligned regions. Following the discovery of the duplication of ST64B genes in Gifsy-1_DT104_ sequence this sequence was also aligned with both ST64B_DT64_ and ST64B_DT104_ sequences. Regions of the Gifsy-1_DT104_ sequence which failed to align with any of the prophage sequences were extracted. In the same way Gifsy-1 sequences were extracted from the genome sequences for DT2, SL1344, D23580 and ATCC 14028. The Gifsy-1 sequence from *S. enterica* subsp *enterica* Serovar 4,[5], 12:i:- strain CVM23701 was extracted from contigs ABAO01000016.1 and ABAO01000067.1 from whole genome shotgun sequencing project ABAO00000000.1 BLASTn was used to compare the various Gifsy-1 sequences, and regions which appeared to be unique to SL1344, DT2 and CVM23701 were extracted. (The sequences for D23580 and ATCC 14028 were not significantly distinct from that of DT2).

The co-ordinates for the Gifsy-3 prophage in ATCC 14028 (1284562 and 1335445) were obtained by aligning the LT2 genome sequence with a sequence from the ATCC 14028 genome estimated to contain the prophage knowing its approximate location from the genome map [21]. Gifsy-2 from LT2 was downloaded using the published co-ordinates [22]. BLASTn was used to locate and extract a region in Gifsy-3 which did not appear in Gifsy-2 or any of the Gifsy-1 sequences. Primers were chosen for the amplification of a portion of this sequence (Table 2). A visual comparison of the Gifsy-1 sequences for LT2, DT104, DT2, SL1344 and CVM23701 and the Gifsy-3 sequence from ATCC 14028 was made using MAUVE [20].

### MLVA Typing

For routine MLVA typing the procedure of Lindstedt et al [18] was followed except for minor modifications: the concentration of primers for STTR6 and STTR9 was halved, the size marker was labelled with ROX and fragment sizing was performed on an ABI 3130 sequencer. MLVA profiles were expressed as a number string of allele sizes (as generated by the sequencer) for each VNTR in the order STTR9-STTR5-STTR6-STTR10pl-STTR3. Additional *S.* Typhimurium VNTRs were amplified using primers shown in Table 2. PCR procedure and product detection were as described below for CRISPR typing except that PCR conditions of 30 cycles of 94°C for 30 s, 57°C to 58°C for 30 s and 72°C for 60 s to 90 s with a final 72°C for 7 min were applied and for STTR1, STTR7, STTR8, STTR11 and STTR12 5% DMSO was included in the mastermix to eliminate non-specific product formation.

### CRISPR Typing

The two CRISPR sequences in each isolate were amplified using a number of primer pairs depending on the size and spacer composition of the CRISPR sequence present (Table 2). For alleles up to 1500 bp adequate amplification was usually obtained using primer pairs spanning the full CRISPR but for longer sequences two PCR products were generated using internal primers derived from spacer sequences. The mastermix contained 2 mM MgCl_2_, 5 pmol of each primer (Geneworks, Adelaide, South Australia) and 0.5 U of AmpliTaq Gold (Applied Biosystems, Foster City, Calif.); the initial cycling step was 95°C for 10 min followed by 30 cycles of 94°C for 15 s, 55°C for 30 s and 68°C for 2.5 m with a final 68°C for 7 m ; a 6 µl aliquot from each PCR tube was electrophoresed in a 1.5% agarose gel containing 0.5 µg/ml ethidium bromide at 80 V for 60 min. PCR products were sequenced using the Applied Biosystems Big Dye Terminator v3.1 and Applied Biosystems 3130 sequencer. The sequences were analysed by ChromasPro. The CRISPR finder program (http://crispr.u-psud.fr/) was used to locate the regular repeats and the intervening spacer sequences. Results were represented as filled rectangular blocks for ‘spacer present’ or an X for ‘spacer absent’ in the same order as for *S.* Typhimurium spacers in Fabre et al [23].

### Amplification of Phage Loci

For amplification of SB6, SB26, SB28, SB37 and SB46 loci in ST64B_DT64_ prophage the primer pairs used were those of Ross and Heuzenroeder [8]. Table 2 shows primers pairs chosen for amplification of seven loci in ST64B_DT104_ which had low or no identity with the ST64B sequence from DT2, of five loci in the Gifsy-1_DT104_ with no identity with either Gifsy-1_LT2_ or ST64B sequence, of six loci in Gifsy-1_SL1344_ and three loci in Gifsy-1_DT2_ with no identity in other Gifsy-1 sequences, of one locus in Gifsy-1_LT2_ shared by Gifsy-1_SL1344_ and Gifsy-1_DT2_ but not by Gifsy-1_DT104_ or Gifsy-2, three loci in Gifsy-1_CVM23701_, two with no identity in other Gifsy-1 sequences and one shared with all Gifsy-2 sequences and with Gifsy-1_LT2_, and one locus in Gifsy-3_14028_. There are also primers for a complex 22/39 bp tandem repeat which occurs in Gifsy-2 but also in Gifsy-1_CVM23701_, where the repeat sequence is longer. The mastermix composition was the same as for CRISPR typing. For amplification the annealing temperature was between 55 and 60°C for 30 s and extension took place at 72°C for 30 to 60 s. Products were visualised on gel and representative ones were sequenced as required.

### Phage Typing

Isolates were sent to the Microbiological Diagnostics Unit, University of Melbourne, Australia for phage typing by the Anderson scheme and antibiotic sensitivity testing.

## Supporting Information

Figure S1 VNTR/CRISPR 1 genotypes for panel of well characterised *S.* Typhimurium strains ordered into Major Groups and Repeats Groups (RG) based on VNTR (MLVA allele string STTR9-STTR5-STTR6-STTR10pl-STTR3+allele sizes for six other VNTR) and CRISPR 1 typing (solid block = spacer present, X = spacer absent). The spacer positions are same as those applied by Fabre et al [34]. The 74 bp spacer includes Spacer 10. Spacer 22 contains a 6 bp tandem repeat Green = 3 repeats, Black = 4 repeats, Red = 5 repeats, Blue = 6 repeats.
(XLS)

Figure S2 VNTR/CRISPR 2 genotypes for panel of well characterised *S.* Typhimurium strains ordered into Major Groups and Repeats Groups (RG) based on VNTR (MLVA allele string STTR9-STTR5-STTR6-STTR10pl-STTR3+allele sizes for six other VNTR) and CRISPR 2 typing (solid block = spacer present, X = spacer absent. The orange block indicates a SNP variant of spacer 10 as per Table 6 in [34]). The spacer positions are same as those applied by Fabre et al [34].
(XLS)

Figure S3 Diagrammatic comparison (not to scale) of the ST64B DNA sequences of *S.* Typhimurium strains DT2 (Sanger) and DT104 (NCTC 13348) showing regions of comparative identity interspersed with regions of non-identity.
(XLS)

Text S1 The DT104-specific SB26 sequence in earlier isolates of DT197.
(DOC)

Text S2 Variation in the ST64B_DT64_ SB46 sequence for the three phage types in RG14.
(DOC)

Text S3 Possible origin of the SB46 variant found in the isolates in RG6B.
(DOC)

Text S4 Alignment of Gifsy-1_DT104_ with ST64B_DT64_ and ST64B_DT104_.
(DOC)

Text S5 Alignment of Gifsy-1 sequences.
(DOC)

Text S6 The tandem repeat for SB42 in isolate 06P23331584.
(DOC)

Text S7 Variation in the 24 bp VNTR in Gifsy-1_DT2_ among Repeats Groups.
(DOC)

Text S8 Supporting evidence for the identification of ST64B sequences in Gifsy-1_DT64_ and Gifsy-1_DT104_.
(DOC)

Text S9 The separation of Major Groups 1 and 2 based on VNTR data.
(DOC)
