# Supplementary material for: Distribution of Gifsy-3 and of Variants of ST64B and Gifsy-1 Prophages amongst Salmonella enterica Serovar Typhimurium Isolates: Evidence that Combinations of Prophages Promote Clonality
Source: PLoS One. 2014 Jan 24;9(1):e86203. doi: 10.1371/journal.pone.0086203 (PMC3901673; doi:10.1371/journal.pone.0086203)
Supplement: Text S1 — The DT104-specific SB26 sequence in earlier isolates of DT197. (DOC) [file pone.0086203.s004.doc]

**Text S1.** Since DT197 first appeared in Australia in 2000 and within five years became one of the most common phage types in Queensland we wished to know if earlier isolates also had the unique SB26 sequence identified in more recent isolates. We chose 38 isolates with a representative variety of MLVA profiles from years 2000 to 2005 (including the only isolate for 2000) for amplification of the 585bp product. All 38 isolates were positive. Product from eight isolates was sequenced. All sequences were identical to the previously sequenced 585bp products except for three isolates from years 2000, 2003 and 2004 which had the same single SNP at position 340. It was notable that the STTR10pl alleles for these three isolates were the rarely seen sizes of 351 and 357bp not present in the other sequenced isolates.
