# Supplementary material for: Distribution of Gifsy-3 and of Variants of ST64B and Gifsy-1 Prophages amongst Salmonella enterica Serovar Typhimurium Isolates: Evidence that Combinations of Prophages Promote Clonality
Source: PLoS One. 2014 Jan 24;9(1):e86203. doi: 10.1371/journal.pone.0086203 (PMC3901673; doi:10.1371/journal.pone.0086203)
Supplement: Text S2 — Variation in the ST64BDT64 SB46 sequence for the three phage types in RG14. (DOC) [file pone.0086203.s005.doc]

**Text S2.** There were differences for the SB46 sequence among the three phage types in RG14. All six of the DT64 isolates had the prototype ST64B_DT64_ SB46 sequence while four out of six DT8 isolates had the prototype ST64B_DT64_ sequence, one isolate had the one SNP sequence and another had an additional 4 SNPs, and two out of six DT9 isolates had the prototype ST64B _DT64_ sequence and four isolates had the one SNP sequence.
