# Supplementary material for: Distribution of Gifsy-3 and of Variants of ST64B and Gifsy-1 Prophages amongst Salmonella enterica Serovar Typhimurium Isolates: Evidence that Combinations of Prophages Promote Clonality
Source: PLoS One. 2014 Jan 24;9(1):e86203. doi: 10.1371/journal.pone.0086203 (PMC3901673; doi:10.1371/journal.pone.0086203)
Supplement: Text S3 — Possible origin of the SB46 variant found in the isolates in RG6B. (DOC) [file pone.0086203.s006.doc]

**Text S3.** Only 4 of the 14 SNPs (relative to SB46 of ST64B_DT104_) in the SB46 variant found in the isolates in RG6B were common to the SB46 sequence in Gifsy-1_DT104_. Since the Gifsy-1 sequence in these isolates was not Gifsy-1_DT104_ it was concluded that the SB46 variant was located on the ST64B_DT104_ prophage. There is evidence that the 14 SNP variant of SB46 allele has been acquired from another serovar. BLASTing of available sequenced Salmonella strains with the 14 SNP variant of SB46 showed that the closest alignment was obtained with serovar Mississippi 2010K-1406, only one SNP removed, and with serovar Weltevreden HI_N05-537 which was 7 SNPs removed. Significantly in these serovars there was total alignment for the five bases 225 to 229 of the SB46 variant allele whereas none of these bases aligned with the SB46 allele in other ST64B sequences nor in Gifsy-1_DT104_ except for ST64B prophage in Enteritidis P125109 where only base 225 was misaligned. In Choleraesuis SC-B67 there is a SB46 allele with a nearly identical sequence to the one in Enteritidis and with the same sequence for bases 225 to 229 as Enteritidis but it is in a Gifsy prophage in the same location as Gifsy-3 in sequenced strain Typhimurium ATCC 14028. We have assessed that in Choleraesuis SC-B67 there is a ‘Gifsy-3’ prophage of 50134bp with coordinates 1306969 and 1357102. Approximately 50% of its sequence is shared with Gifsy-1_DT104_ but only 44% with Gifsy-3_14028_.
