# Supplementary material for: Distribution of Gifsy-3 and of Variants of ST64B and Gifsy-1 Prophages amongst Salmonella enterica Serovar Typhimurium Isolates: Evidence that Combinations of Prophages Promote Clonality
Source: PLoS One. 2014 Jan 24;9(1):e86203. doi: 10.1371/journal.pone.0086203 (PMC3901673; doi:10.1371/journal.pone.0086203)
Supplement: Text S4 — Alignment of Gifsy-1DT104 with ST64BDT64 and ST64BDT104. (DOC) [file pone.0086203.s007.doc]

**Text S4.** Alignment of Gifsy-1_DT104_ and ST64B_DT64_ showed three regions of similarity corresponding to: the last 173bp of the 37-38 intergenic region through to the start of SB42 at high level of identity except for part of SB41 with much lower identity; the last 74bp of SB42 through to the end of SB48 at 97% identity; and the start of SB52 through to a little less than half of SB53 at 90% identity. Alignment of Gifsy-1_DT104_ and ST64B_DT104_ also showed three regions of similarity. Two were very similar to the first two above and the other was most of the 50-51 intergenic region through most of SB52 at only 64% identity. This included the SB52 region of ST64B_DT104_ which showed 66% identity with ST64B_DT64_. In contrast the Gifsy-1 sequences from the other strains showed no significant similarity to either ST64B_DT64_ or ST64B_DT104_.
