# Supplementary material for: Distribution of Gifsy-3 and of Variants of ST64B and Gifsy-1 Prophages amongst Salmonella enterica Serovar Typhimurium Isolates: Evidence that Combinations of Prophages Promote Clonality
Source: PLoS One. 2014 Jan 24;9(1):e86203. doi: 10.1371/journal.pone.0086203 (PMC3901673; doi:10.1371/journal.pone.0086203)
Supplement: Text S5 — Alignment of Gifsy-1 sequences. (DOC) [file pone.0086203.s008.doc]

**Text S5.** Alignment of Gifsy-1_DT2_, Gifsy-1_D23580_ and Gifsy-1_14028_ showed almost no significant differences except for different numbers of repeats for the STTR6 tandem repeat and an additional 5bp repeat in Gifsy-1_14028_ at position 22137 not present in the other strains. Alignment of Gifsy-1_LT2_ and Gifsy-1_DT104_ showed that there were three regions of sequence difference: a 1294bp sequence located between 19638 and 20931 on Gifsy-1_LT2_ but absent in Gifsy-1_DT104_ and corresponding to the location of the *GipA* virulence gene; an 856bp sequence located between 29105 and 29960 on Gifsy-1_DT104_ but absent in Gifsy-1_LT2_; and non-identity for most of the sequence between 30346 and 42334 on Gifsy-1_DT104_ encompassing virtually all of the sequence which contains the ST64B sequences. A very similar result was seen when Gifsy-1_DT104_ was aligned with each of Gifsy-1_DT2_, Gifsy-1_D23580_ and Gifsy-1_14028_. Alignment of Gifsy-1_SL1344_ and Gifsy-1_DT104_ showed a similar result for the three regions of sequence difference but there were two additional regions of difference located between 42500 and 43310 and between 43829 and 44369 on Gifsy-1_DT104_ downstream from the 3’ end of the third region. Alignment of Gifsy-1_SL1344_ and Gifsy-1_DT2_ showed the same two additional regions of difference as seen with Gifsy-1_SL1344_ and Gifsy-1_DT104_ but there were significantly more regions of identity. Between 37319 and 44527 on Gifsy-1_SL1344_ there were four regions of sequence difference interspersed with regions of identity.
