# Supplementary material for: Distribution of Gifsy-3 and of Variants of ST64B and Gifsy-1 Prophages amongst Salmonella enterica Serovar Typhimurium Isolates: Evidence that Combinations of Prophages Promote Clonality
Source: PLoS One. 2014 Jan 24;9(1):e86203. doi: 10.1371/journal.pone.0086203 (PMC3901673; doi:10.1371/journal.pone.0086203)
Supplement: Text S6 — The tandem repeat for SB42 in isolate 06P23331584. (DOC) [file pone.0086203.s009.doc]

**Text S6.** Isolate 06P23331584 in RG3 (Figure S1) or RG4 (Figure S2) with a unique MLVA type and a unique STTR12 allele had a sequence corresponding to the substitute sequence for SB42 of ST64B which had a tandem repeat with three 15bp repeats instead of only two as seen in all the other sequences.
