# Supplementary material for: Distribution of Gifsy-3 and of Variants of ST64B and Gifsy-1 Prophages amongst Salmonella enterica Serovar Typhimurium Isolates: Evidence that Combinations of Prophages Promote Clonality
Source: PLoS One. 2014 Jan 24;9(1):e86203. doi: 10.1371/journal.pone.0086203 (PMC3901673; doi:10.1371/journal.pone.0086203)
Supplement: Text S7 — Variation in the 24 bp VNTR in Gifsy-1DT2 among Repeats Groups. (DOC) [file pone.0086203.s010.doc]

**Text S7.** The 24bp VNTR in Gifsy-1_DT2_ from DT2 has 3.8 repeats but in all the DT135a sequences from Whole Genome Shotgun projects at DDBJ/EMBL/GenBank the 24bp VNTR in Gifsy-1_DT2_ has only 1.8 repeats. Amplification of the VNTR locus in isolates belonging to Repeats Groups with the Gifsy-1_DT2_ prophage (Table 3) using primers shown in Table 1 showed that 81out of 84 isolates from RGs 1, 12A, 12B and 12C had the 3.8 repeats the same as Gifsy-1_DT2_ while three isolates from RG12B had 2.8 repeats. All 129 isolates from RGs 12D, 13A, 13B and 13C had 1.8 repeats the same as the sequenced DT135a isolates.
