# Supplementary material for: Distribution of Gifsy-3 and of Variants of ST64B and Gifsy-1 Prophages amongst Salmonella enterica Serovar Typhimurium Isolates: Evidence that Combinations of Prophages Promote Clonality
Source: PLoS One. 2014 Jan 24;9(1):e86203. doi: 10.1371/journal.pone.0086203 (PMC3901673; doi:10.1371/journal.pone.0086203)
Supplement: Text S8 — Supporting evidence for the identification of ST64B sequences in Gifsy-1DT64 and Gifsy-1DT104. (DOC) [file pone.0086203.s011.doc]

**Text S8.** Tucker and Heuzenroeder have shown that when the digested DNA preparations of isolates of phage types DT9, 64, 12a and 197 were probed with the PCR amplicon from the *immC* region of ST64B they produced a band on a >8.51-kb fragment which from our results we can now identify as being located on Gifsy-1_DT64_ and Gifsy-1_DT104_. We have not tested for the *immC* genes in our testing because they are shared by Gifsy-1_DT104_ and ST64B but the results of Tucker and Heuzenroeder confirm that other shared sequences are present in the Gifsy-1 prophages from these phage types. However, we know that the identification of the prophage variant based on the assumption outlined here needs to be modified for isolates which have both Gifsy-1 and Gifsy-3 prophages since it would lead to the false conclusion that they contain two different Gifsy-1 prophages, Gifsy-1_DT2_, and Gifsy-1_SL1344_ which shares all of its ‘unique’ sequences with Gifsy-3.
