# Supplementary material for: Distribution of Gifsy-3 and of Variants of ST64B and Gifsy-1 Prophages amongst Salmonella enterica Serovar Typhimurium Isolates: Evidence that Combinations of Prophages Promote Clonality
Source: PLoS One. 2014 Jan 24;9(1):e86203. doi: 10.1371/journal.pone.0086203 (PMC3901673; doi:10.1371/journal.pone.0086203)
Supplement: Text S9 — The separation of Major Groups 1 and 2 based on VNTR data. (DOC) [file pone.0086203.s012.doc]

**Text S9.** Most isolates in Major Group 1 have an STTR3 allele of 456, 462, 489, 516, or 543bp, an STTR7 allele of 620, 582, 505 or 346bp and an STTR9 allele of 171, 180, 189 or 198bp (rarely 162bp) while most isolates in Major Group 2 have an STTR3 allele of 370, 456, 496, 523 or 550bp, an STTR7 allele of 612, 380 or 543bp (rarely 582) and an STTR9 allele of 162bp (rarely 171bp). Sequencing of the various STTR3 and STTR7 alleles has shown that there are nucleotide substitutions in the sequences of the repeats as well as different repeat lengths and arrangements. The comparison of allele sequences provides very suggestive evidence that the sets of STTR3 and STTR7 alleles in Major Group 1 have followed a different evolutionary pathway from the corresponding sets in Major Group 2 (results not shown).
